# The Molecular Architecture for the Intermediate Filaments of Hard **α**-Keratin Based on the Superlattice Data Obtained from a Study of Mammals Using Synchrotron Fibre Diffraction

**DOI:** 10.1155/2011/198325

**Published:** 2011-10-19

**Authors:** Veronica James

**Affiliations:** Research School of Chemistry, Australian National University, Canberra, ACT 0200, Australia

## Abstract

High- and low-angle X-ray diffraction studies of hard **α**-keratin have been studied, and various models have been proposed over the last 70 years. Most of these studies have been confined to one or two forms of alpha keratin. This high- and low-angle synchrotron fibre diffraction study extends the study to cover all available data for all known forms of hard **α**-keratin including hairs, fingernails, hooves, horn, and quills from mammals, marsupials, and a monotreme, and it confirms that the model proposed is universally acceptable for all mammals. A complete Bragg analysis of the meridional diffraction patterns, including multiple-time exposures to verify any weak reflections, verified the existence of a superlattice consisting of two infinite lattices and three finite lattices. An analysis of the equatorial patterns establishes the radii of the oligomeric levels of dimers, tetramers, and intermediate filaments (IFs) together with the centre to centre distance for the IFs, thus confirming the proposed helices within helices molecular architecture for hard **α**-keratin. The results verify that the structure proposed by Feughelman and James meets the criteria for a valid **α**-keratin structure.

## 1. Introduction

Despite 70 years of investigations into the internal and external organization of the intermediate filaments (IFs) of *α*-keratin, there still remains a wide disparity of opinions published in a wide range of journals regarding the architecture of these IFs. Samples for fibre diffraction experiments are normally mounted so that the X-ray beam is perpendicular to the direction of growth of the specimen. All linear lattices within the sample, aligned in this direction or capable of projection onto it, will contribute to the meridional diffraction pattern obtained [1], providing the lattice spacing falls within the resolution of the equipment. The lattice periodicity *d* for each lattice can be determined by Bragg's law. 

In 1983 and in subsequent papers [2–4] Fraser and MacRae suggested that both a finite lattice of 19.79 nm and a dislocated infinite lattice of 47.00 nm could be determined from X-ray diffraction. However, Lawrence [5] reported that this did not satisfy all known data. Further lattices 19.8 nm, 27.2 nm, 12.2 nm, and 7.8 nm were added by Wilk et al. [6] and James et al. [7]. However, there still remained 4 quite large unaccounted-for reflections which subsequently were shown to increase in intensity for hair from insulin-dependent diabetic (IDDM) humans and IDDM baboons, James et al. [8]. As a result the presence of a further infinite lattice of 62.6 nm was confirmed bringing the total visible lattices to two infinite lattices and 3 finite lattices with two further possible lattices (7.8 nm and 15.6 nm) submerged. A possible molecular assembly of all these lattices for human hair was proposed by Feughelman and James [9] for echidna quill, Lincoln wool, and horse hair. This was extended to human hair by James and Amemiya [10] and for pathological hair specimens [8, 11]. Mathematical confirmation combined with results from mechanical properties further confirmed this proposed structure [12]. 

**Figure 1:**
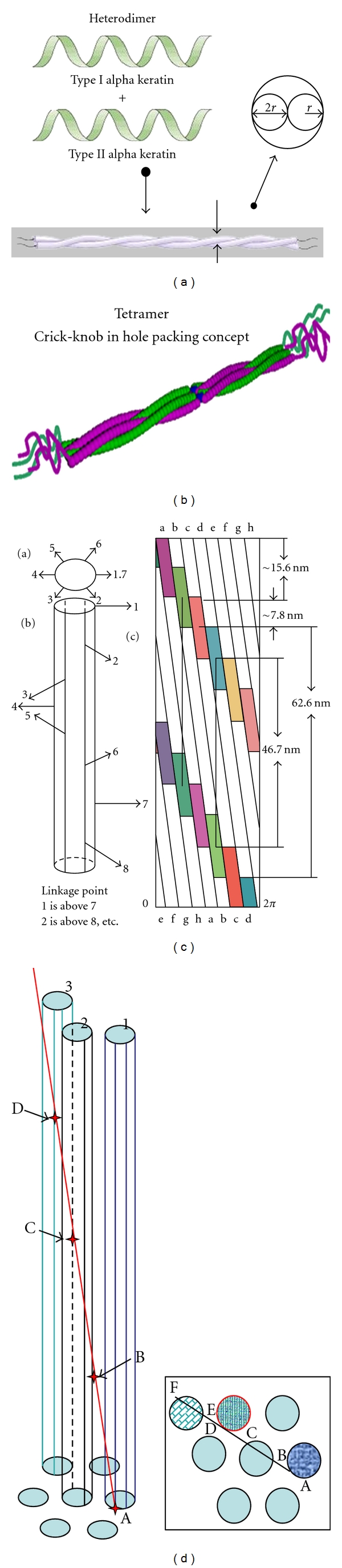
(a) This figure shows the winding together of two hetero keratin fibrils to form a dimer. (b) This figure shows the winding together of two dimers in a Crick “knob in hole” packing. (c) This figure depicts the lattices that superimpose on the diffraction pattern resulting in the superlattice that is the resultant of the superposition of the seven lattices discussed below. The 47 nm lattice is associated with the distance between the beginning of the helical section of one tetramer and the beginning of the helical section of the next but one tetramer, for example, AB, BD, thus creating an obvious infinite and continuous lattice in the direction of the hair. The finite lattices (19.8 nm, 272 nm, and 12.4 nm), recorded by Wilk et al. [6], James et al. [7], Feughelman et al. [12], and James [11] are subsets of the projections of the 47 nm lattice being the projection of the 200 nm section of the helical section [15] plus and minus the nonhelical section [8, 11, 12]. All reflections from the two other lattices 7.8 nm and 15.6 nm representing the C and N terminal noncoiled ends and their sum [15] are buried under reflections of the other lattices. The repeat distance of 62.6 nm is the projection on the direction of the fibre of the complete tetramers, that is, the projection of the sum of the lengths of the helical and nonhelical sections of the tetramer, for example, AC, as it winds through 120°. Considering a line entering at A and leaving at C, the presence of an infinite lattice with spacing 62.6 nm is not so obvious. As the geometrical analysis shows [10] it is mathematically possible for one and only one condition, namely, that the distance between the IFs is three-times the radius of the IF. This is illustrated in Figure 2 where A, B, C, D, and E are successive points along the lattice. They are separated by ~63.1 nm which projects onto the direction of the hair as 62.6 nm. The insert is a view vertically downwards showing a sequence of lattice points of this infinite lattice traversing through the hexagonal array as it progress along the hair. (d) This figure shows the progress of the infinite 62.6 nm lattice as it progresses the length of the hexagonal array of intermediate filaments (IFs) in the sample. The unique hexagonal geometrical arrangement of the IFs, namely, that the centre to centre spacing of adjacent IFs is three-times the radius of the IF, and the fact that there is 60° between the linkage points on adjacent tetramers, give rise to an infinite repeating lattice, one set of points being A, B, C, D, E, and so forth, that has a projected spacing of 62.6 nm in the direction of the hair. The vertical view shows the sideways progression across the hexagonal array of successive points on the lattice as it moves along the length of the sample. Each point is separated along the length of the hair by 62.6 nm from its neighbours.

This helices within helices model is shown in Figure 1 as the final result of two hetero keratin helices winding together to form a tight dimer (Figure 1(a)), the radius of which is twice the radius of that of the single helices. Two of these dimers then wind together to form a tetramer, using a Crick “knob in hole” packing, [13], (Figure 1(b)). Eight tetramers then wind in a staggered array around the surface of the intermediate filaments. The repeat distance of 62.6 nm, is the projection on the direction of the fibre of the complete tetramers that is the projection of the sum of the lengths of the helical and nonhelical sections of the tetramer, for example, AC, as it winds through 120°.  Figure 1(c) shows the final set of lattices. 

Considering a line entering at A and leaving at C, the presence of an infinite lattice with spacing 62.6 nm is not so obvious, especially with the well-known hexagonal packing. As the geometrical analysis shows [10] this is mathematically possible for one and only one condition, namely, that the distance between the IFs is three-times the radius of the IF. This is illustrated in Figure 1(d) where A, B, C, D, and E are successive points along the lattice. They are separated by ~63.1 nm which projects onto the direction of the hair as 62.6 nm. The insert is a view vertically downwards showing a sequence of lattice points of this infinite lattice traversing through the hexagonal array as it progresses along the hair. 

The following discussion is based on the hypothesis that this architectural arrangement is not only valid for these samples but also holds universally across a wide spectrum of hard alpha keratin tissues.

## 2. Materials and Methods

### 2.1. Samples

Single human, horse, cow, goat, dog, cat, elephant (*Elephas maximus*), baboon (*Papio hamadryas*), and orangutan (*Pongo pygmaeus abelii*) hairs were cut from near the skin and were analysed without any special chemical preparation except for a short cleaning with distilled water prior to mounting in cells specially designed to hold the hairs taut without stretching. An age series of hairs from cows was included together with hairs being taken from the tail and also the ear. Bundles of 20 or more fur fibres cut from near the skin of a rock wallaby (*Petrogale lateralis*), a northern koala (*Petrogale lateralis*), a western grey kangaroo (*Macropus fuliginosus*), and a red kangaroo (*Macropus rufus*) together with bundles of alpaca wool were carefully aligned in parallel arrays, tied together at the ends, and mounted in the specially designed cells so that the every hair in the bundle was taut and not stretched. Small sections of rhinoceros horn (*Rhiniceros unicornis*), horse hoof, and fingernails approximately 3 mm by 0.9 mm were cut in the direction parallel to the growth of the *α*-helices. These sections were inserted into and fixed to the ends of glass capillaries approximately 1 mm internal diameter so that the sample to be examined protruded from the ends. Porcupine quills (*Porcupine hystrix indica*) and echidna quills (*Tachyglossus aculeatus*) were also mounted into the ends of glass capillaries so that their tips protruded for irradiation with X-rays. These capillaries were held in place in specially grooved plates during data collection. The highly uniform wool chosen for analysis was taken from Lincoln sheep that had been penned and fed under controlled conditions for uniformity of growth and designated SW 296 [14]. Since there was little or no statistical variation along the length of this wool, single fibres could be used for this study. 

All samples were collected in accordance with the ethics policies of the respective zoos and the experiments carried out in accordance with the ethics and safety policies of the respective synchrotrons and research laboratories. The transportation and exportation of and experimentation with all Cites and native Australian samples were approved by the Australian Government Department of Environment and Water Resources. The importation of samples to the United States was approved by the TSA of USA.

### 2.2. X-Ray Scattering Setup and Collection of Data

**Figure 2:**
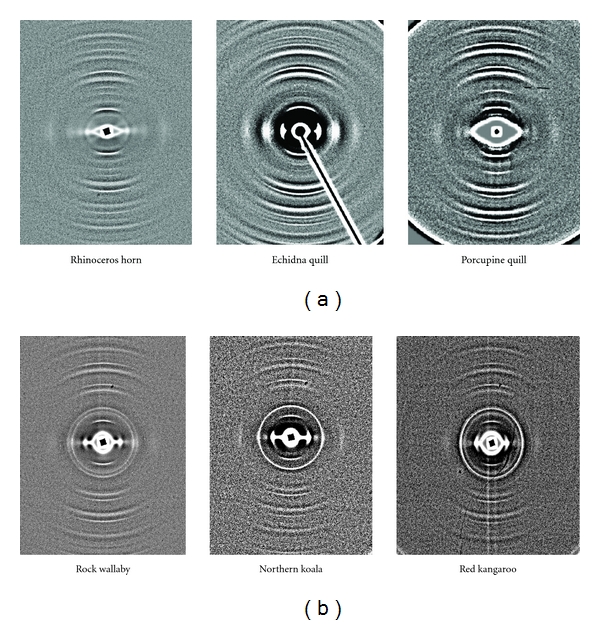
(a) Typical diffraction patterns obtained from small sections of rhinoceros horn and from the tips of porcupine and echidna quills using a beam centrally located on the sample at the BioCAT facility. (b) Typical diffraction patterns obtained at the BioCAT facility for bundles of parallel fur fibres from Australian marsupials. These samples were found to be poorly diffracting and required thirty or more parallel strands to be irradiated to give reliable results.

Two-dimensional X-ray diffraction patterns were obtained using the synchrotron X-ray source at the Advanced Photon Source (Argonne National Laboratory) on both the BioCAT and ChemMatCARS beamlines. On the BioCAT beamline, the X-ray energy was 120 keV, the distance of the sample to MAR165 detector based on the second-order diffraction ring in the scattering pattern from Silver Behenate {*d*-spacing = 58.380(5)} was 1048.5 mm. Fuji Bas III imaging plates, located perpendicular to the incident beam and in front of the detector were also used on the BioCAT beamline. The pixel size of the image plates was 0.1 × 0.1 mm. Three or more samples of each species were investigated on each of these beamlines. The beam size was 100 *μ*m in the horizontal direction and 50 *μ*m in the vertical direction so all samples were mounted with the growth direction of the *α*-keratin in the horizontal direction and the sample centred in the beam. Samples were moved backwards and forwards along their lengths as it is important to locate the optimum position along the sample and then moved up and down to ensure that the beam is located centrally within the sample. This was especially important for the horn, hooves, nails, and quills and for the marsupial fur where single hair patterns were too weak to be meaningful and so bundles of parallel fibres had to be used. Typical examples of the diffraction pattern as obtained on BioCAT for the horns and quills are given in Figure 2(a) and for the marsupial fur bundles in Figure 2(b).

**Figure 3:**
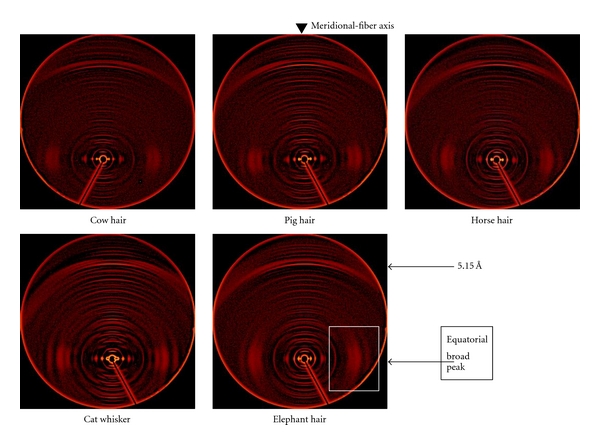
Typical off-centre diffraction patterns of 5 mammals taken on ChemMatCARS showing meridional reflections extending beyond the 5.15 nm reflection, the 91st order of the 46.7 nm lattice. These patterns reflect the wealth of diffraction from such keratin samples.

Because of the limitation on the size of the output imposed by the dimensions of the detectors or imaging plates and the evacuated flight tube, the latter being essential to remove absorption of the weak diffracted beam, the very strong 5.15 Å meridional reflection was missed. To allow an extended view, after diffraction patterns similar to those above were obtained, the detector on ChemMatCARS was moved off centre and the wider diffraction patterns taken again in this position. Some examples of these for hair and whiskers background removed by SAX15ID are shown in Figure 3. 

Smoothing of the data and background removal were achieved using combinations of a number of different techniques, FIT2D and SAX15ID, MATLAB and ProcessFITS, and IRAF and SAO. The first and third of these used a background removal process designed in my laboratory as the most efficient method in the analysis of keratin, reported by Wilk et al. [6]. In this procedure, the data is smoothed as in Figure 6.

Background removal using the combination of MATLAB and Process was effected by taking 20 or more points along the curve and finding the best polynomial fit for these points. The results were very similar for all the patterns analysed to those obtained from the IRAF and SAO packages. Intensity plots from strips 10 mm on either side of the vertical and horizontal axes were also obtained using IRAF and the positions and intensities of all peaks from each of these plots were read and recorded.

## 3. Results

### 3.1. Meridional Pattern

The *meridional* pattern for any fibre diffraction pattern is the superposition of the diffraction patterns of all lattices finite and infinite in the direction along the fibre giving rise to a superlattice when more than one lattice is involved. In any superlattice, the intensities of the various points along the lattice are the additions of the wave contributions of all the lattices or the resultant of both the phases and intensities. The resultant intensities can be reduced to very low values especially if the scattering factors are similar or the phase differences are *π* radians out of phase. The intensities are only large if all the maxima coincide at that point. The meridional pattern of hard *α*-keratin has very few strong reflections. The strongest of all the low angle peaks is that at 67 Å. This peak is the resultant of the 7th order maximum of the 46.7 nm lattice, the third order maximum of the 19.67 nm lattice, the second order of the 12.4 nm lattice, and the fourth order maximum of the 27.2 nm with the ninth order maximum of the 62.6 nm lattice near the base of the peak closest to the pattern centre causing a bulge on the peak. The high-intensity 5.15 Å reflection is also a combination of the maxima of all these lattices including the 91st order of the 46.7 nm lattice, the 39th-order maximum of the 19.67 nm lattice, the 26th order of the 12.4 nm lattice and the 52nd-order maximum of the 27.2 nm with the 121st order maximum of the 62.6 nm lattice near the base of the peak on the lower angle edge. However, the 14th, 18th, 22nd, and 47th lattice points of the 62.6 nm lattice are unique and quite strong in all patterns. 

Most of the remaining peaks are quite weak but there are sufficient medium intensity unique orders of each lattice to quickly verify the presence of the lattice. In looking for the weak orders that were present, it was essential to differentiate between true peaks and noise. To this purpose the patterns for the same sample in the same position were taken for 10 seconds, 20 seconds, and 30 seconds and plotted on the same graph. If the reflection is a true lattice point, then the intensity should increase with increase in exposure time. If it does not then it must be dismissed as noise. Using this criterion for the selection of true lattice points, the position of all peaks for all samples was located. The total number of orders present for the 46.7 nm lattice and the 62.6 nm lattice are recorded in Table 1. 

Some individual extra reflections are visible in different groups of animals such as marsupials and whiskers but these are superimposed on the keratin structure and may relate to samples or other use of whiskers. These are not discussed here.

### 3.2. Equatorial Pattern

**Figure 4:**
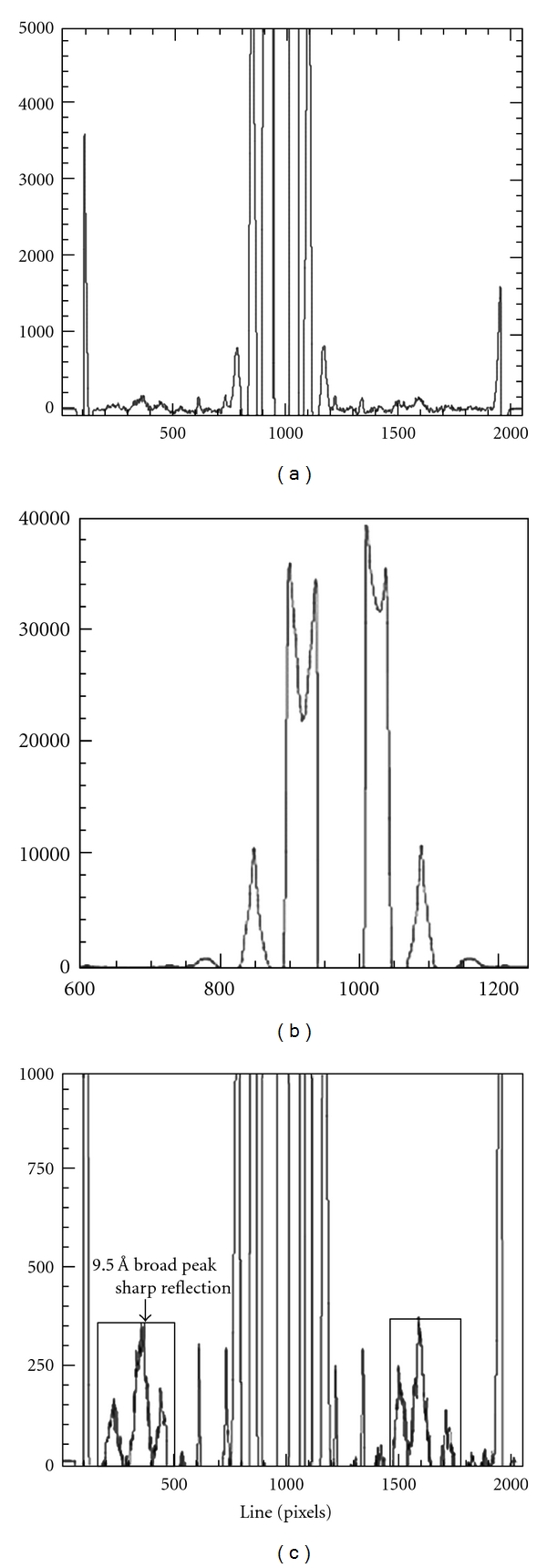
These three patterns are background-corrected equatorial plots, taken over 1 mm on either side of the meridional axis. (a) shows the complete plot showing sets of  “spots” and arcs. There were usually 9 spots, the one nearest the centre illustrated in (b) giving the centre to centre distance between the IFs. The other “spots” are the various orders of the Bessel functions from which the IF and tetramer (protofibril) radii can be calculated. In most cases there were six orders available to determine the IF radii and 2 for the tetramer radii. For most samples there were 8 arcs which index onto a spacing of ~45 Å and are related to the presence of soaps [16] and one sharp arc. The sharp arc, indicated in (c), verifies the close binding of the alpha-helices forming the heterodimer since the centre to centre distance of the two alpha-helices is equal to the diameter of each single helix.

A plot of the intensity distribution in the *equatorial* direction after background removal, using a cut 1 mm wide on either side of the axis for a catwhisker is given in Figure 4(a) with the centre detail given in the Figure 4(b) and the detail of the broad 9.5 Å peak in Figure 4(c). These postbackground correction X-ray equatorial profiles (perpendicular to the length of the hair) were very well resolved for all samples including the area of the broad 9.5 Å peak. As can be seen from Figures 4(a) and 4(c) this broad peak is the resultant of three diffuse peaks and one sharp arc. This sharp arc is present in all samples and has been regarded by all investigators as the result of interference between the tightly coiled *α*-keratin polypeptide chains verifying the centre to centre distance between the *α*-keratin helices is approximately equal to the diameters of the individual helices [6, 17–19].

As can be seen from the corresponding diffraction pattern in Figure 3, the maxima in Figure 4 correspond to the series of dots and arcs in the diffraction pattern. Up to 8 orders of the arcs are present in the samples examined. These arcs, which index onto a spacing of ~45 Å, were initially attributed to crystallised lipids but later identified more precisely as soaps (Briki et al. [16]). Since the arcs are not related to the *α*-keratin architecture, they fall outside the scope of this study.

**Figure 5:**
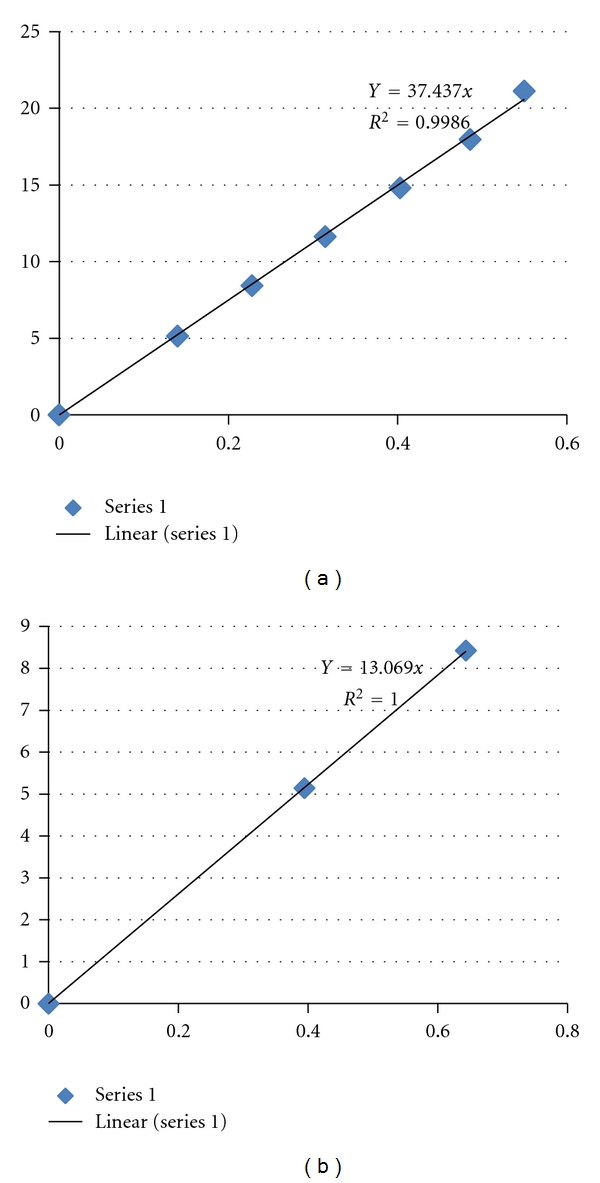
These are typical examples of the first-order Bessel function graphs used to determine the radii of the IFs (a) and the tetramers (b). The *y*-axis represent the Bessel functions, the *x*-axis represents *X* = *n*_*p*_/*d*_*c*_ *n*_*c*_, where *n*_*p*_ is the pixel value of the relevant peak, *d*_*c*_ = 46.7 nm, *n*_*c*_ = pixel value for 46.7 nm spacing. The slope of the line of best fit for each graph gives the required radius whilst *R* ^2^ is the statistical error in fitting the line to the actual peaks.

**Figure 6:**
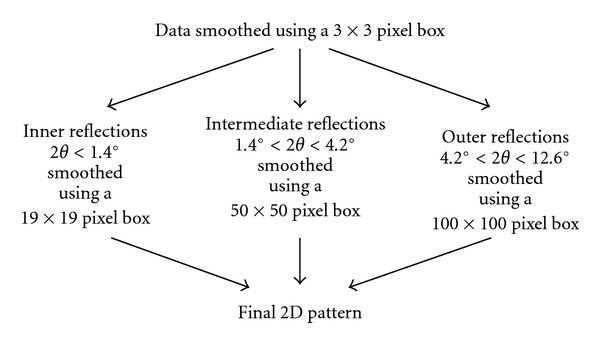


The positions of the six to nine clearly visible spots on the equatorial intensity plots were recorded for each sample. The radii of the IFs, the tetramers, and the centre to centre spacings of the IFs were determined assuming sets of solid cylinders using a Bessel function analysis [6, 20–22]. The results obtained for each of these parameters for all samples are given in Table 2. The graphs of the Bessel function calculations of the IF radius and the tetramer radius for the fine hair of the 50-year-old elephant are given in Figures 5(a) and 5(b), respectively. In these graphs the Bessel function (*y* axis) is plotted versus 2*πX* (*x* axis) where *X* = *n*_*p*_/*d*_*c*_ *n*_*c*_, *n*_*p*_ is the pixel value of the relevant peak, *d*_*c*_ = 46.7 nm, *n*_*c*_ = pixel value for 46.7 nm spacing.  Figure 5(a) is the graph for the calculation of the IF radius and Figure 5(b) is the graph for the calculation of the tetramer radius. The slope of the graph gives the required radius in each case and *R* ^2^ the accuracy.

## 4. Discussion and Conclusion

The results in Table 1 confirm the presence of the two infinite lattices of the superlattice for all samples. For samples mounted centrally, although the fourth order was the first order resolvable, 42 ± 3 orders of the 46.7 nm lattice and 56 ± 2 orders of the 62.6 nm lattice were present for all samples studied at APS, 50 orders of the 46.7 nm and 72 orders of the 62.6 nm for samples studied at the Photon Factory. When the samples were positioned off centre on ChemMatCARS so as to record higher orders, 119 orders of the 46.7 nm lattice and 158 orders of the 62.6 nm lattice were recorded. The peaks for these lattices were identified in series of plots along the meridional axes. The 4 : 3 relationships between these two lattice parameters give some superposition of these reflections but three quarters of the 62.6 nm peaks were unique to this lattice and resolvable. These results verify absolutely the presence of a 62.6 nm infinite lattice for all samples.

The results obtained from the Bessel function analysis of the equatorial intensity distribution are listed in Table 2. These results indicate very accurate values for the IF radii, the tetramer radius, and the distance apart of the helices. Other lattices also appeared in the mouse and cat whisker samples but were not common to other samples, so, have not been included in this analysis. The results verify that the required relationship between the radius of the intermediate filaments and the distance apart of the intermediate filaments required to validate the proposed architecture of the *α*-keratin also holds. The average value obtained for this ratio for the 27 samples was 3.0, standard deviation 0.04. These results verify the second requirement for this structure.

Since all results that are present in the fibre diffraction patterns must relate to any proposed architectural arrangement for the *α*-keratin intermediate filaments, the presence of the 62.6 nm lattice and the fact that the distance apart of the IFs is 3-times the IF radius must be explicable by any proposed structure in addition to the accepted 46.7 nm lattice and subsets of this lattice. The results presented here indicate that the model proposed by Feughelman and James in 1998 based on mechanical experimental results and fibre diffraction results certainly meets these criteria.

## Figures and Tables

**Table 1:** Number of orders present for each of the two infinite lattices.

Sample	Number of orders	
	46.7 nm	62.6
Alpaca wool	42	58
Baboon hair	50	72
Cat whisker	114	155
Cat fur	42	58
Cow hair	118	158
Dog hair	43	59
Echidna quill	41	54
Elephant fine hair	40	55
Elephant coarse hair	40	54
Goat beard	40	54
Goat hair	39	54
Horse hair	115	153
Horse hoof	39	54
Human hair	50	72
Red kangaroo hair	42	57
White kangaroo hair	42	56
Koala hair	40	54
Lincoln wool	40	55
Orangutan	39	53
Pig hair	39	57
Porcupine quill	40	54
Rhinoceros	114	154
Rock wallaby	44	58

**Table 2:** Equatorial results.

Animal	*Scientific name *	Sample	Age	IF radius (nm)	B!	*R* ^2^	Tetramer radius (nm)	B!	*R* ^2^	IF-IF	Number of samples		*(IF-IF)/IF*
											BC	CMC	
Alpaca	*Lama pacos*	Wool bundle		38.2	4	0.999	13.1	2	0.999	114 ± 2	1	2	2.98
Asian elephant	*Elephas maximus*	Hair thick	50 y	37.2	4	0.998	12.6	2	0.999	111 ± 3	1	1	2.98
Asian elephant	*Elephas maximus*	Hair thin	50 y	37.4	6	0.998	13.1	2	1	113 ± 5	1	1	3.02
Baboon 1	*Papio hamadryas*	Hair		37.3	4	0.999	12.7	2	0.994	112 ± 3	1	1	3.00
Baboon 2	*Papio hamadryas*	Hair		38.8	4	0.999	13.0	2	0.999	117 ± 2	1	1	3.01
Cat	*Felis domesticus*	Whisker	2.5 y	36.0	4	1	13.0	2	0.999	109 ± 3	1	1	3.02
Cat	*Felis domesticus*	Hair	22 y	38.9	3	0.999	13.2	2	1	116 ± 2	1	1	2.98
Calf	*Bos taurus*	Head hair	1 m	37.7	3	0.999	13.0	2	1	112 ± 2	1	0	2.97
Cow moppet	*Bos taurus*	Hair from ear	2-3 y	37.7	4	0.999	12.9	2	0.999	112 ± 2	1	1	2.97
Cow moppet wide	*Bos taurus*	Tail hair	2-3 y	35.8	4	0.998	12.8	2	0.998	106 ± 3	1	1	2.96
Cow goldie	*Bos taurus*	Top head hair	4 y	38.1	4	0.999	12.8	2	1	115 ± 4	2	2	3.02
Dog	*Canis lupus*	Hair	6 m	37.9	4	0.999	12.8	2	1	112 ± 6	2	2	2.96
Echidna	*Tachyglossus aculeatus*	quill		37.4	4	0.999	13.3	2	1	111 ± 3	2	2	2.98
Goat	*Capra hircus*	Beard		37.5	4	0.999	13.0	2	0.999	114 ± 4		1	3.04
Goat	*Capra hircus*	Head hair		36.6	5	0.999	13.6	2	0.999	111 ± 3		1	3.03
Horse	*Equus caballus*	Hoof	22 y	37.9	4	0.999	13.5	2	0.999	115 ± 3	2	1	3.03
Horse	*Equus caballus*	Tail hair	22 y	34.9	4	0.998	13.1	2	0.999	104 ± 4	1	2	2.98
Kangaroo, red	*Macropus rufus*	Fur bundle	1–10 y	38.3	4	0.999	13.0	2	0.999	116 ± 3	1	2	3.03
Kangaroo, Western Grey	*Macropus fuliginosus melanops *	Fur bundle	3–11 y	38.6	4	0.999	13.2	2	0.999	113 ± 6	15	2	2.93
Northern koala	*Phascolarctos cinereus*	Fur bundle	8–13 y	37.5	3	1	13.5	2	0.999	113 ± 4	1	2	3.01
Orang-utan	*Pongo pygmaeus abelii*	Hair	32 y	39.1	4	1	13.2	2	0.999	114 ± 2	1	1	2.92
Orang-utan	*Pongo pygmaeus abelii*	Hair	37 y	40.1	4	0.999	12.9	2	0.999	121 ± 3	2		3.06
Pig	*Sus scrofa*	Hair from ear		35.5	4	0.999	12.4	2	0.999	107 ± 3	1	1	3.01
Porcupine	*Porcupine hystrix indica*	Quill		36.9	4	0.998	12.6	2	0.999	111 ± 2	1	2	3.00
Rhinoceros	*Rhiniceros unicornis*	Horn		35.4	4	0.998	12.8	2	0.999	107 ± 3	3	1	3.04
Rock wallaby	*Petrogale lateralis*	Hair bundle	4 y	38.9	4	0.999	12.4	2	0.999	119 ± 2	1	0	3.06
Sheep	*Ovis aries*	Wool		37.9	4	0.999	13.1	4	1	114 ± 5	13	2	3.01
										Average = 3.00			
										Standard Deviation = 0.04			

B! number of Bessel Points, *BC: BioCAT, CMC: ChemMatCars.*
